# Dietary Patterns and Oral Health Behaviours Associated with Caries Development from 4 to 7 Years of Age

**DOI:** 10.3390/life11070609

**Published:** 2021-06-24

**Authors:** Cátia Carvalho Silva, Sandra Gavinha, Sofia Vilela, Rita Rodrigues, Maria Conceição Manso, Milton Severo, Carla Lopes, Paulo Melo

**Affiliations:** 1 Faculdade de Medicina Dentária, Universidade do Porto (U. Porto), Rua Dr. Manuel Pereira da Silva, 93, 4200-393 Porto, Portugal; pmelo@fmd.up.pt; 2 Faculdade de Ciências da Saúde, Universidade Fernando Pessoa, Praça 9 de Abril, 349, 4249-004 Porto, Portugal; sgavinha@ufp.edu.pt (S.G.); cmendes@ufp.edu.pt (R.R.); cmanso@ufp.edu.pt (M.C.M.); 3 EPIUnit-Instituto de Saúde Pública da Universidade do Porto, Rua das Taipas, 135, 4050-600 Porto, Portugal; sofia.vilela@ispup.up.pt (S.V.); milton@ispup.up.pt (M.S.); carlal@med.up.pt (C.L.); 4 Environmental and Health Research Unit (FP-ENAS), Universidade Fernando Pessoa Energy, 4249-004 Porto, Portugal; 5 Associated Laboratory for Green Chemistry (LAQV/REQUIMTE), Universidade do Porto, 4050-313 Porto, Portugal; 6 Departamento de Biomedicina, Faculdade de Medicina, Universidade do Porto, Alameda Prof. Hernâni Monteiro, 4200-319 Porto, Portugal

**Keywords:** dental caries, early childhood caries, dietary patterns, oral health behaviours, children, paediatric dentistry, oral public health

## Abstract

The association between modifiable risk factors and caries in children has been documented; however, most studies have been cross-sectional and have not considered the complexity of dietary factors and oral health habits. This study aimed to investigate the prospective association between dietary patterns and oral health behaviours at 4 years of age and the development of new decayed, missing, and filled teeth (d_3–6_mft/D_3–6_MFT) over a period of three years. Participants were children from the Generation XXI population-based birth cohort. At 4 years of age, diet patterns were assessed using a food frequency questionnaire, with three dietary patterns being identified. For the purpose of capturing the new development of caries between 4 and 7 years of age, two dental outcomes were defined in the mixed dentition: “dental caries development” and “severe dental caries development” in the mixed dentition. Bivariate analysis and multivariate logistic regression were used. From 4 to 7 years of age, 51.2% of the children had at least one new d_3–6_mft/D_3–6_MFT and 27.4% had more than two new d_3–6_mft/D_3–6_MFT. Children belonging to the “energy-dense foods” (OR = 2.19; 95% CI: 1.20–4.00) and “snacking” (OR = 2.19; 95% CI: 1.41–3.41) dietary patterns at 4 years old were associated with severe dental caries development three years later. Preventive strategies should be implemented in an attempt to reduce snacking and the consumption of energy-dense, micronutrient-poor foods to promote children’s oral health.

## 1. Introduction

Dental caries is one of the most prevalent chronic diseases in paediatric age, with an estimated 621 million children worldwide having untreated caries lesions in the primary dentition [1].

A systematic review with a meta-analysis, which was recently published in order to determine the prevalence of this disease in primary and permanent dentition in children from different continents, showed that dental caries affects 46.2% and 53.8% of children in primary and permanent dentition, respectively [2]. Due to its worrying incidence and other characteristics that are related to its pathophysiological process, dental caries continues to represent an important public health problem in paediatric age [3].

Dental caries is attributed to both modifiable risk factors, such as dietary factors, fluoride exposure, and toothbrushing frequency; and non-modifiable risk factors, such as socioeconomic status and previous caries experience [4]. 

Dietary and oral health behaviours have been most commonly reported as risk factors for caries among children [5]; however, there is still little evidence on which factors have strong associations with the disease [6].

There is abundant evidence that sugar consumption by children has an influence on caries experience [7]. In March 2015, the World Health Organization (WHO) published a new guideline on sugar intake for adults and children [8]. A strong recommendation was made for both children and adults that the intake of free sugars should be reduced to ≤10% of the total energy intake. To protect oral health throughout the lifecourse, the WHO also made a conditional recommendation for a further reduction of the intake of free sugars to below 5% of total energy intake [7,8,9,10]. Nowadays, when considering the amount versus frequency of sugar intake, the findings suggest that the frequency and the texture of foods seem to fit better with the current understanding of the pathophysiological mechanism of caries [11]. Concerning this issue, Goodwin and colleagues reported that the intake of free sugar snacks during the day was not found to predict caries experience; however, the consumption of these snacks before bedtime may be an important risk factor for this dental disease [12]. Additionally, a recent systematic review suggested that restricting free sugars before and at bedtime may reduce the risk of caries in children [13]. 

Children do not consume a single nutrient or food group, and investigating dietary patterns has the advantage of examining the overall diet, including the foods, food groups, and nutrients, as well as their combination and variety [14]. It is possible to miss out on the effects of and interaction with other potentially contributory groups when examining only a single food group or nutrient for its impacts on a disease [15]. Examining dietary patterns in children may provide more information on the effects of dietary factors on the incidence of dental caries than the examination of sugar intake alone [15,16].

Toothbrushing is considered a fundamental self-care behaviour for maintaining oral health [17]. Many studies have found that toothbrushing performed twice or more times per day increases the effectiveness of fluoride toothpaste in reducing the incidence of caries [18,19]. Nevertheless, the effects of toothbrushing frequency on the prevention of dental caries is unclear. Besides the toothbrushing frequency, other toothbrushing-related factors such as the fluoride content of the toothpaste, the amount of toothpaste used [20], the time spent brushing the teeth [21], and toothbrushing supervision [22,23] can interfere with the anticaries effects of toothbrushing in children; therefore, the relationship between children’s toothbrushing frequency and the incidience of dental caries is worth investigating, with a greater emphasis on performing studies with strict methodologies regarding factors related to toothbrushing in order to enable possible cause–effect relationships to be determined [24].

To develop effective prevention strategies for dental caries, an understanding of how caries develops and progresses over time is necessary; however, few large longitudinal cohort studies have been completed in children [25].

Traditionally, observational caries studies have been cross-sectional in design, which can be suggestive of risk factors for disease but are limited in terms of establishing relationships between exposure and outcome over time. Only a few prospective studies so far have dealt with the question of which modifiable risk factors are correlated with caries in children. In particular, there have been few longitudinal studies bridging the transitional period between early and later childhood. Comparing between 4 and 7 years of age, there is a great difference in the exposure times of the primary and permanent teeth to behavioural factors; therefore, this period of tooth transition is crucial in order to accurately assess the impacts of modifiable risk factors on dental caries experiences in children. 

The aim of this study was to investigate the prospective association between dietary patterns and oral health behaviours at 4 years of age and the development of new decayed, missing, and filled teeth in later childhood (at 7 years of age). 

## 2. Materials and Methods

### 2.1. Study Design and Participants

Participants are part of the Generation XXI, an ongoing prospective population-based birth cohort [26]. Briefly, a total of 8647 children and their mothers were recruited between April 2005 and August 2006 from all five public maternity clinics in the metropolitan area of Porto, Portugal. All cohort participants from baseline were invited to participate at the 4-, 7-, and 10-year follow-ups, whereby 86%, 80%, and 76% of the children were re-evaluated, respectively. As part of the physical evaluation, the children were also invited for an oral examination at 4 and 7 years of age, with 5226 and 910 children, respectively, receiving dental examinations. 

The present study comprised a sample of children evaluated at baseline and at 4 and 7 years of age. The 619 children included were from the 910 total children that participated in the oral examination at 7 years of age. We excluded children with diseases that might influence dental caries and dietary intake (cerebral palsy, celiac disease, food allergy, food intolerance, phenylketonuria, congenital malformations, diabetes mellitus, lymphoma or leukaemia; n = 12), leaving a final sample of 607 children. The characteristics of the participants who attended the dental visit and of their mothers were compared with those of the remaining non-participating cohort evaluated at baseline. In our study, the monthly household income was higher than in the remaining cohort (45.2% vs. 40.1%, *p* = 0.022). No significant differences were found for each child’s sex and weight at birth, gestational age, and the mother’s age and education level (Table 1).

The Generation XXI project was conducted according to the Declaration of Helsinki. The Ethical Committee of the São João Hospital and the Faculty of Medicine of the University of Porto approved all procedures involving human subjects or patients. The Portuguese Authority of Data Protection also approved this study. Legal representatives of each participant received an explanation on the purpose and design of the study and gave written informed consent at the baseline and follow-up evaluations.

### 2.2. Data Collection

Data at birth and at both 4 and 7 years of age were collected by trained interviewers in face-to-face interviews, through structured questionnaires that included information on the mothers’ socioeconomic and demographic characteristics and the children’s health history and lifestyle factors. The variables from the baseline evaluation used in the present study included the following: the child’s sex (male or female) and the mother’s age (continuous variable) and education level (≤9 y, 10–12 y or >12 y). Household income was assessed at the 4-year follow-up.

### 2.3. Dietary Intake

Children’s food consumption information was collected through a food frequency questionnaire (FFQ), which had been previously tested and calibrated [27] and was administered by trained interviewers. At the 4-year follow-up, the parents or caregivers were asked how many times on average in the previous 6 months their child had consumed 35 food items. 

All consumption frequencies (ranging from “never” to “≥4 times per day”) were converted into daily frequencies as previously described [28]. Two food groups were defined under two new variables, called “cariogenic foods” (ice cream, breakfast cereals, crackers, cookies, sweet pastry, chocolate, sugar, and candies) [9] and “cariogenic drinks” (sweetened carbonated drinks and other sugar-sweetened drinks) [29], and the consumption was considered as the frequency per day. Both these variables were analysed as continuous variables.

As described earlier [30], to identify dietary patterns of children at 4 years of age, a total of 16 food items or groups were defined (i.e., milk, yoghurt, meat and eggs, sugar-sweetened beverages, sweets, fruit, vegetable soup, vegetables on plate, cheese, fish, processed meat, rice/pasta/potatoes, bread, crisps, pizza/burgers, and salty pastry). Three dietary patterns were identified by latent class analysis (LCA) [30]: “healthier”, “snacking”, and “energy-dense foods” (EDF). The number of patterns were defined according to the Bayesian information criteria (BIC) and children were assigned to each pattern according to the highest probability of class membership. The “healthier” pattern was characterised by higher consumption of fruit, vegetables, and fish, and consumption of EDF. The “snacking” pattern was defined by a low consumption of foods typically consumed at main meals and an intermediate to high consumption of snacking foods (e.g., milk, yoghurt, sweets, salty pastry, and crisps). The EDF pattern was characterised by higher consumption of energy-dense, micronutrient-poor food items, such as sweets and sugar-sweetened beverages; and high-fat foods, such as pizza, burgers, processed meat, and salty pastry [30].

### 2.4. Dental Examination

The clinical dental examinations at 4 (CDE1) and 7 years of age (CDE2) comprised a dental evaluation for the children and a questionnaire for their legal guardians on toothbrushing frequency and other dental-health-related behaviours. The children’s toothbrushing scores were converted into two groups: <2 times per day or ≥2 times per day, none at bedtime; and ≥2 times per day, one at bedtime. Other oral health-related behaviours included: whether the child had parental-supervised toothbrushing (no/yes); whether the child used fluoridated toothpaste (no/yes); if the child had already attended a dental appointment (no/yes); and whether the child eats at bedtime, after toothbrushing (no/yes).

CDE1 was carried out by a team of six dentists and ten master’s and PhD students in dentistry, who were responsible for collecting data from clinical observations and for the dental interviewing procedures. For CDE1, children were assessed in the dental chair with their teeth clean and dry, using a mirror and a probe. At CDE2, the oral evaluation was performed by four dentists. The participants were examined in a standard chair with a halogen lamp, using a dental mirror and a probe.

At CDE1 and CDE2, the intra- and inter-examiner calibration consisted of a session that included an e-learning programme, a theoretical course with images, and a training observation of patients of the same age against a gold standard. To assess consistency between observations, each examiner repeated one out of every ten clinical observations during the recording stage. At the 4-year follow-up, the dentists’ intra- and inter-examiner calibration scores showed linear weighted kappa values of 0.75 and 0.70, respectively, for the International Caries Detection and Assessment System II (ICDAS II), with a good agreement on both [31]. Similarly to CDE1, at CDE2 a new team of four dentists (except for one examiner from the previous examination) achieved a moderate-to-high agreement for ICDAS II (linear weighted kappa values of 0.80 and 0.75, respectively) [31].

At each clinical dental examination, caries lesions were recorded on a surface-related level according to the ICDAS II criteria [32]. To obtain dmft/DMFT scores (decayed, missing, and filled teeth), the number of carious teeth in the primary and permanent teeth were counted for each child using the following categories: sound surfaces (ICDAS II 00, 0A, 10, 1A, 20, 2A), decayed surfaces (ICDAS II 03–06, 13–16, 23–26, 33–36, 43–46, 53–56, 63–66, 70–76, 83–86), filled surfaces (ICDAS II 30, 3A 40, 4A 50, 5A 60, 6A, 80, 8A), and missing tooth surfaces due to caries (ICDAS II 97). At the tooth level, if a tooth had a decayed surface and another tooth was filled, the tooth was considered to be decayed. 

In this analysis, only the cavitated lesions were considered (d_3–6_mf/D_3–6_MF ICDAS II codes 3–6). No additional detection methods, including radiographs, were used. Each tooth was examined individually and the condition was recorded. 

The “dental caries status” was defined as the presence or absence of cavitated dental caries in primary and mixed dentition for 4- and 7-year-old children (d_3–6_mft = 0 vs. d_3–6_mft > 0 and d_3–6_mft/D_3–6_MFT = 0 vs. d_3–6_mft/D_3–6_MFT > 0, respectively). The proportion of individuals that developed new cavitated caries and missing and filled teeth between CDE1 and CDE2 was calculated. Using these 2 summary scores separately, each participant was further categorised into caries-free and caries-active groups to calculate caries prevalence.

For the purpose of capturing the new development of caries in the mixed dentition, two main outcome variables were created for caries. The first was “dental caries development” (DCD) (having ≥ 1 new cavitated caries, missing and filled teeth: yes/no) and the second dental outcome was “severe dental caries development” (S-DCD) (having > 2 new cavitated caries, missing and filled teeth: yes/no) in mixed dentition between CDE1 and CDE2. 

### 2.5. Statistical Analysis

Statistical analysis was performed using the statistical software package IBM SPSS® Statistics vs. 26.0 (IBM Corp. released 2017, IBM Corp., Armonk, NY, USA). Descriptive statistical analysis was applied and categorical variables were depicted by frequencies and proportions or percentages (n and %), while quantitative variables were expressed as median and interquartile range (IQR: 1st and 3rd quartiles). For inferential statistics, the level of significance was set at *p* < 0.05. The comparison of two groups regarding the median values of quantitative variables was performed using the Mann–Whitney U test, due to the fact that those variables presented a non-normal distribution (lack of symmetry in most cases). Bivariate analysis of qualitative variables was performed using the chi-square test and all pairwise comparisons were adjusted using Bonferroni correction. The confidence intervals (CI; 95% confidence level) of the prevalence of caries at CDE1 and at CDE2 and of the incidence of caries from 4 to 7 years of age were calculated using the exact method.

Further multivariate logistic regression analyses (crude and adjusted) were performed to evaluate crude and adjusted odds ratio (OR) and 95% CIs for DCD and S-DCD. Variables with *p* < 0.20 (in the previously mentioned bivariate analysis) regarding DCD and S-DCD were included in the first step of the model. Exceptions for the variables “daily frequency of cariogenic foods intake” and “daily frequency of cariogenic drinks intake” were excluded due to the evidence of multicollinearity with dietary patterns. For theoretical reasons [13], the use of fluoridated toothpaste was included in the models. Backward stepwise Wald elimination was performed (*p* < 0.05 for covariate inclusion and *p* > 0.20 for exclusion). The models were adjusted for the child’s sex and mother’s age and education level in years. Additionally, the dietary patterns were adjusted to toothbrushing frequency and to eating before going to bed and after toothbrushing.

## 3. Results

The caries prevalence rates were 27.5% (95% CI: 24.0–31.3) at CDE1 and 54.2% (95% CI: 50.1–58.2) at CDE2, with 14.5% (95% CI: 11.8–14.6) of participants already showing cavitated caries lesions at 7 years in the permanent teeth. From 4 to 7 years of age, 51.2% (95% CI: 47.2–55.3) of the children had at least one new decayed, missing, or filled tooth and 27.4% (95% CI: 24.3–31.6) had more than two new decayed, missing, or filled teeth.

Table S1 presents the children’s characteristics for the sample according to dental caries status at 4 and 7 years of age. At both ages, mothers of children with dental caries had significantly lower schooling years compared to mothers of caries-free children (4 y: 49.1% vs. 40.4%, *p* = 0.004; 7 y: 50.5% vs. 33.8%, *p* < 0.001). Similarly, children with caries had lower monthly family incomes. Regarding oral health behaviours, children in both clinical dental examinations mostly performed toothbrushing two or more times per day, including one at bedtime with fluoride toothpaste, but only at 7 years of age were significant statistical differences found in terms of dental caries status (*p* = 0.040). On the other hand, children with caries had no parental supervised toothbrushing (4 y: 82.9% vs. 64.4%, *p* < 0.001; 7 y: 88.3% vs. 93.8%, *p* = 0.022). Additionally, more caries were observed in children following a dietary pattern rich in energy-dense foods (4 y: 51.5 vs.43.5%, *p* = 0.048; 7 y: 51.7% vs. 38.8%, *p* = 0.002), and on average the children’s daily frequency of cariogenic drink consumption was higher compared to children with no caries (4 y: median: 0.98 (IQR: 0.42–1.98) vs. 0.67 (IQR: 0.26–1.26), *p* = 0.001; 7 y: median 0.87 (IQR: 0.34–1.48) vs. 0.65 (IQR: 0.26–1.26) (Table S1).

The results for the association of DCD and S-DCD from 4 to 7 years of age with exposure variables at 4 years are presented in Table 2.

Both dental outcomes were associated with mothers who completed <10 schooling years (DCD: 51.8% vs. 33.4%, *p* < 0.001; S-DCD: 60.0 vs. 33.4%, *p* < 0.001), with a monthly household income ≤ 1000 € (DCD: 35.0% vs. 19.1%, *p* < 0.001; S-DCD: 40.1% vs. 25.8%, *p* < 0.001), and eating before going to bed (DCD: 47.3% vs. 37.7%, *p* = 0.017; S-DCD: 51.2% vs. 37.7%, *p* = 0.005). No parental-supervised toothbrushing and children belonging to the “snacking” pattern were associated with DCD (11.7% vs. 6.5%, *p* = 0.029 and 14.4% vs. 12.6%, *p* = 0.002, respectively). On the other hand, belonging to “EDF” pattern at 4 years of age was associated with S-DCD in 7-year-old children (53.7% vs. 39.5%, *p* < 0.001) (Table 2).

In the crude analysis, a positive association was found between eating before going to bed and after toothbrushing and S-DCD at 7 years of age. The adjustment for child’s sex and mother’s age and education level did not attenuate the association between this dietary behaviour (eating before going to bed) and S-DCD (OR = 1.77; 95% CI: 1.15–2.74). Compared to the “healthier” pattern, children belonging to the “EDF” pattern had higher odds of DCD (OR = 1.77; 95% CI: 1.24–2.53) and S-DCD (OR = 2.19; 95% CI: 1.41–3.41) after adjustment for potential confounders (child’s sex, mother’s age and education, toothbrushing frequency, and eating before going to bed). Additionally, the multivariate logistic regression analysis indicated a significant adjusted association between the “snacking” pattern and S-DCD (“snacking” pattern vs. “healthier” pattern: OR = 2.19; 95% CI: 1.20–4.00) (Table 3).

## 4. Discussion

This longitudinal study, which collected comprehensive data at the individual level, helps in better understanding the aetiological factors of the development of new dental caries with detailed information about each child’s dietary intake and their oral health behaviours. 

The present results showed that after adjustment for potential confounders, children belonging to the “EDF” and “snacking” dietary patterns at 4 years of age and those who ate at bedtime after toothbrushing had a significant risk of S-DCD at 7 years of age. The “EDF” dietary pattern was also a significant risk factor for DCD at the same age. In this sample, the factors related to diet at 4 years old seemed to be of higher relevance for later caries development than those specifically related to oral health care, such as toothbrushing.

It is widely believed that mechanical disruption of cariogenic biofilm is one of the cornerstones of caries prevention [18,19]; however, higher toothbrushing frequency, specifically at bedtime, was not associated with lower caries experience in this longitudinal analysis. Some studies have reported an inverse relationship between toothbrushing frequency and caries experience in children [33,34,35], while others have found no association [36]. The evidence base seems weak, especially when it comes to toothbrushing frequency [18]. Toothbrushing per se is associated with several factors that can affect the results. In an attempt to overcome these drawbacks, in this analysis we included other toothbrushing-related factors, such as the use of fluoride toothpaste, toothbrushing before going to bed, and parental supervised-toothbrushing. The inclusion of these variables allowed us to obtain reliable results.

The significant relationship between eating before going to bed and caries incidence reported in this study is consistent with previous studies [6,12,37]; however, this association raises two important issues: (1) bedtime intake may be part of a general pattern of increased free sugar intake [12]; or (2) bedtime intake per se, regardless of sugar intake during the day, may be the most important determinant of caries incidence. During sleep, the salivary flow decreases markedly, reducing the self-cleansing effect and buffering capacity of saliva in the oral cavity [38]. This condition contributes to the disturbance of the oral balance in the direction of demineralisation, increasing the risk of dental caries [39].

Our results showed that children following the “EDF” and “snacking” patterns, when compared with those belonging to the “healthier” pattern, respectively showed higher odds of S-DCD and DCD later in life. High consumption of energy-dense, micronutrient-poor foods and beverages such as sweets and sugar-sweetened beverages, characteristics of the “EDF” dietary pattern, may help explain the positive association with new caries development in 4–7-year-old children. This association was also observed in previous studies with similar characteristics.

In the scientific literature, higher added-sugar beverages have been identified as a stronger predictor of new dental caries than cariogenic foods in childhood [16,35,40,41]. In our study, the consumption of cariogenic foods and beverages was not shown to be associated with the development of new caries lesions in the three years of follow-up. This may be due to the way that cariogenic food and beverage groups are defined, as well as characteristics of their intake that were not recorded (e.g., as snacks, at meals, at bedtime).

Interestingly, other previous studies with younger children did not find an association between consumption of cariogenic food and drinks and dental caries [42,43]; however, our results demonstrated that the higher added-sugar beverages were associated with a higher prevalence of caries at 4 and 7 years of age. 

The “snacking” dietary pattern was characterised by an intermediate to high consumption of certain foods between the main meals, which promotes the continuous availability of carbohydrates to the oral cavity. These nutrients sources provide substrates for bacterial acid production, which causes local pH values in the dental biofilm to fall, favouring sustained demineralisation of dental tissues. Frequent drops over time in the pH of the dental plaque may explain the positive association between the “snacking” dietary pattern and S-DCD.

Dietary patterns at age 4 were associated with the development of caries at age 7, because in a previous analysis it was found that children’s dietary patterns at 4 years of age were maintained in school-age children (7 years) and because dental caries is a cumulative disease.

In Portugal, the National Food, Nutrition, and Physical Activity Survey (IAN-AF 2015–2016) reported a free sugar mean daily intake of 50 g per day in children aged 5 to 9 years old. The main dietary sources of free sugars were yoghurts and sweets (foods commonly consumed as snacks), with lower intake levels in children with more educated parents [44]; thus, it is crucial to advise patients and populations that total intake of free sugars should be reduced as part of a holistic intervention for oral health.

At 7 years of age, the proportion of caries-free children in our sample was similar to the proportion of 6-year-old children reported in the northern region of Portugal in the Third National Study on Oral Diseases (33.8% 95 CI: 28.0–40.3) held in between 2012 and 2014 [45,46]; however, it seems that the WHO’s 2020 goal for this age group of having 80% of children caries-free will not be achieved [47]. Despite the preventive efforts that have been made in the last few decades, there has still been a significant increase in dental caries in children. The high incidence of caries observed highlights the need for comprehensive longitudinal studies with strict methodologies.

In the present study, the associations between dietary patterns, oral health behaviours, and new caries development were approached prospectively, allowing the establishment of a temporal relationship between these characteristics and enabling certain limitations of the previous studies to be overcome.

In the present study, we did not find an association between consumption of cariogenic food and drinks and new dental caries development during the 3-year follow-up period. This result leading to a reflection on the importance of differentiating their characteristics (definitions of cariogenic food and beverage groups) and intake (e.g., as snacks, at a meal, at bedtime) in future investigations.

Besides the prospective design and the children’s age range, another strength of this study was the follow-up period of 3 years in an important age group as the children transition from the primary to the permanent dentition; in this time interval, the tooth considered most susceptible to caries erupts, namely the first permanent molar. The changes in the social and school environments experienced in these years will influence the risk of caries and have lifelong impacts on oral health.

The caries assessment method was used here to diagnose dental caries at the cavitated stage. This decision was taken because the study was designed to assess a public health domain. Cavitated caries require treatment and have implications for children, their caregivers, and the health care system in terms of costs and capacity. The caries assessment method used is the same one used in local and national disease surveillance programs; therefore, it allows comparisons to be made with recent and future epidemiological findings [25]. Finally, as children of this study were part of a large population-based cohort that has been regularly followed, several potential confounding factors could be assessed.

Some limitations of this study need further discussion. The inclusion of a sub-sample could have introduced bias into the analysis due to the burden related to the timing of the dental examinations. Comparing the children included in our study with the remaining cohort, we did not find statistically significant differences regarding sex, gestational age, weight at birth, mother’s age, or educational level, although the mean family monthly income in this study sample was higher (45.2% vs. 40.1%, *p* = 0.022) in comparison with the mean family monthly income of the remaining cohort; however, this difference was more likely to be due to the large sample size rather than to substantial differences between participants. Despite adjustment for relevant confounders, certain residual confounding factors cannot be excluded. The assessment of dietary intake using an FFQ could also be argued as a limitation; however, comparisons with 3 day food diaries supported the reasonable validity and reliability of the FFQ data [27].

## 5. Conclusions

Children belonging to the “EDF” and “snacking” dietary patterns at 4 years of age were associated with S-DCD at 7 years of age. The present findings suggest the need to implement effective strategies in an attempt to reduce the consumption of snacking and energy-dense, micronutrient-poor foods by children in order to prevent dental caries. From a public health perspective, the development of food policies in countries such as Portugal has been directed towards taxation of sugary beverages and regulation of the availability of foods with high sugar contents in schools and health units, which should be reinforced and continuously promoted [44]. In clinical settings, the importance of promoting a healthier dietary pattern and adequate oral health measures targeted to very young children should be reinforced by dental professionals.

## Figures and Tables

**Table 1:** Comparison between the characteristics of the eligible participants and the remaining cohort evaluated at baseline * (numbers of participants and percentages, with medians and interquartile ranges).

	Sample ^¥^ (n = 607)		Remaining Cohort ^§^ (n = 8040)		
	n	%	n	%	*p*
**Child’s sex n (%)**					
Female	289	47.6	3947	49.1	0.482
Male	318	52.4	4093	50.9	
<37 weeks	39	7.9	484	9.2	0.361
≥37 weeks	452	92.1	5783	90.8	
**Weight at birth** (g)	607		8039		
Median	3240		3190		0.112
(IQR)	(2900–3509)		(2880–3490)		
**Mothers’ age** (years) **n**	607		8034		
Median	29		29		0.446
(IQR)	(26–33)		(25–33)		
**Maternal education** (years) **n**	606		6818		
Median	12		11		0.087
(IQR)	(9–15)		(8–14)		
Low: ≤1000 €	163	27.2	2117	32.0	**0.022**
Intermediate: 1001–1500 €	165	27.5	1849	27.9	
High: >1500 €	271	45.2	2652	40.1	

* For each variable, the total may not add to 607/8040 due to missing data; ^¥^ children who attended the dental examination at 4 and 7 years of age; ^§^ cohort evaluated at baseline; IQR, interquartile range.

**Table 2:** Bivariate analysis of dental caries development and severe dental caries development from 4 to 7 years of age and correlations with independent (exposure) variables.

	Dental Caries Development			Severe Dental Caries Development		
	New Caries = 0 n (%)	New Caries > 0 n (%)	*p*	New Caries = 0 n (%)	New Caries > 2 n (%)	*p*
**Child’s sex**						
Female	146 (49.3)	143 (46.0)	0.410	146 (49.3)	76 (45.8)	0.465
Male	150 (50.7)	168 (54.0)		150 (50.7)	90 (54.2)	
**Maternal education**						
≤9 years	99 (33.4)	160 (51.8)	**<0.001**	99 (33.4)	99 (60.0)	**<0.001**
10–12 years	95 (32.1)	87 (28.2)		95 (32.1)	49 (29.7)	
>12 years	102 (34.5)	62 (20.1)		102 (34.5)	17 (10.3)	
**Monthly household income**						
Low: ≤1000 €	56 (19.1)	107 (35.0)	**<0.001**	75 (25.8)	65 (40.1)	**<0.001**
Intermediate: 1001–1500 €	75 (25.6)	90 (29.4)		81 (27.8)	56 (34.6)	
High: >1500 €	162 (55.3)	109 (35.6)		135 (46.4)	41 (25.3)	
**Toothbrushing frequency**						
<2 or ≥2 times per day, none at bedtime	123 (42.0)	152 (49.8)	0.054	123 (42.0)	83 (50.9)	0.066
≥2 times per day, 1 at bedtime	170 (58.0)	153 (50.2)		170 (58.0)	80 (49.1)	
**Fluoride toothpaste**						
No	14 (6.3)	15 (6.6)	0.907	14 (6.3)	7 (5.9)	0.884
Yes	207 (93.7)	212 (93.4)		207 (93.7)	111 (94.1)	
**Parental-supervised toothbrushing**						
No	19 (6.5)	36 (11.7)	**0.029**	19 (6.5)	19 (11.5)	0.064
Yes	272 (93.5)	272 (88.3)		272 (93.5)	146 (88.5)	
**Dental appointment at 4-year-old**						
No	199 (67.9)	203 (65.3)	0.491	199 (67.9)	112 (67.5)	0.921
Yes	94 (32.8)	108 (34.7)		94 (32.1)	54 (32.5)	
**Eating before go to bed**						
No	180 (62.3)	162 (52.6)	**0.017**	180 (62.3)	80 (48.8)	**0.005**
Yes	109 (37.7)	146 (47.4)		109 (37.7)	84 (51.2)	
**Dietary patterns**						
Energy-dense foods	116 (39.5)	158 (51.8)	**0.002**	116 (39.5)	87 (53.7)	**<0.001**
Snacking	37 (12.6)	44 (14.4)		37 (12.6)	29 (17.9)	
Healthier	141 (48.0)	103 (33.8)		141 (48.0)	46 (28.4)	
**Daily frequency of cariogenic food intake**						
n	213	230	0.575	294	165	0.622
Median (IQR)	2.74 (1.99–3.75)	2.82 (1.85–4.12)		2.39 (1.72–3.42)	3.24 (2.09–4.39)	
**Daily frequency of cariogenic drinks intake**						
n	214	230	0.063	294	165	0.070
Median (IQR)	0.67 (0.26–1.26)	0.855 (0.34–1.48)		1.34 (0.9–2.42)	1.93 (1.24–3.01)	

Bold entries denote statistical significance (*p* < 0.05); data are presented as proportions (%) for categorical measures and median values and interquartile ranges for continuous measures; differences between groups were tested with Fisher’s exact test or chi-square test, respectively.

**Table 3:** Multivariable logistic regression model to predict dental caries development and severe dental caries development in 4- to 7-year-old children.

	Dental Caries Development		Severe Dental Caries Development	
	**Crude OR**	Adjusted OR *	**Crude OR**	Adjusted OR *
Healthier	Ref	Ref	Ref	Ref
Snacking	1.14 (0.66–1.96)	1.47 (0.88–2.46)	1.56 (0.81–3.01)	**2.19 (1.20–4.00)**
Energy-dense foods	1.43 (0.97–2.11)	**1.77 (1.24–2.53)**	1.58 (0.97–2.58)	**2.19 (1.41–3.41)**
**Eating before go to bed**	**Crude OR**	Adjusted OR **	**Crude OR**	Adjusted OR **
Yes	1.31 (0.92–1.88)	1.30 (0.91–1.85)	**1.75 (1.13–2.70)**	**1.77 (1.15–2.74)**

Bold entries denote statistical significance (*p* < 0.05); OR, odds ratio; CI, confidence interval; variable(s) entered on step 1: toothbrushing frequency at 4 years old, the use of fluoridated toothpaste, parental supervised toothbrushing, dental appointment at 4 years old, eating before going to bed, dietary patterns. * Adjusted for children’s sex, maternal age and education, toothbrushing frequency, and eating before going to bed. ** Adjusted for children’s sex, maternal age and education.

## Data Availability

Data used in this study were from the Generation XXI birth cohort, under the responsibility of Professor Henrique Barros, head of the Department of Public Health and Forensic Sciences and Medical Education of the University of Porto Medical School and president of the Institute of Public Health of the University of Porto. In the present study, individual-level information was used, which cannot be disseminated due to confidentiality issues. A formal request to the person responsible (Professor Henrique Barros: hbarros@med.up.pt) can be made by anyone interested in developing scientific research based on data collected within the Generation XXI study. Further information can be found at the Institute of Public Health website: http://ispup.up.pt/research/research-structures/ (accessed on 10 June 2021).

## Supplementary Materials

The following are available online at https://www.mdpi.com/article/10.3390/life11070609/s1. Table S1: Children’s characteristics of the sample according to dental caries experience at 4 years of age (d_3–6_mft = 0 vs. d_3–6_mft > 0) and at 7-years of age (d_3–6_mft/D_3–6_MFT = 0 vs. d_3–6_mft/D_3–6_MFT > 0).

## References

[1] Kassebaum N.J., Bernabé E., Dahiya M., Bhandari B., Murray C.J.L., Marcenes W. (2015). Global burden of untreated caries: A systematic review and metaregression. J. Dent. Res..

[2] Kazeminia M., Abdi A., Shohaimi S., Jalali R., Vaisi-Raygani A., Salari N., Mohammadi M. (2020). Dental caries in primary and permanent teeth in children’s worldwide, 1995 to 2019: A systematic review and meta-analysis. Head Face Med..

[3] Dickson-Swift V., Kenny A., Gussy M., McCarthy C., Bracksley-O’Grady S. (2020). The knowledge and practice of pediatricians in children’s oral health: A scoping review. BMC Oral Health.

[4] Shivakumar S., Srivastava A.C., Shivakumar G. (2018). Body Mass Index and Dental Caries: A Systematic Review. Int. J. Clin. Pediatr. Dent..

[5] Warren J.J., Weber-Gasparoni K., Marshall T.A., Drake D.R., Dehkordi-Vakil F., Dawson D.V., Tharp K.M. (2008). A longitudinal study of dental caries risk among very young low SES children. Community Dent. Oral Epidemiol..

[6] Levine R.S., Nugent Z.J., Rudolf M.C.J., Sahota P. (2007). Dietary patterns, toothbrushing habits and caries experience of schoolchildren in West Yorkshire, England. Community Dent. Health.

[7] Moynihan P.J., Kelly S.A.M. (2014). Effect on caries of restricting sugars intake: Systematic review to inform WHO guidelines. J. Dent. Res..

[8] World Health Organization (2015). Sugars Intake for Adults and Children.

[9] Moynihan P. (2016). Sugars and Dental Caries: Evidence for Setting a Recommended Threshold for Intake. Adv. Nutr..

[10] Moynihan P., Makino Y., Petersen P.E., Ogawa H. (2018). Implications of WHO Guideline on Sugars for dental health professionals. Community Dent. Oral Epidemiol..

[11] Van Loveren C. (2019). Sugar Restriction for Caries Prevention: Amount and Frequency. Which Is More Important?. Caries Res..

[12] Goodwin M., Patel D.K., Vyas A., Khan A.J., McGrady M.G., Boothman N., Pretty I.A. (2017). Sugar before bed: A simple dietary risk factor for caries experience. Community Dent. Health.

[13] Baghlaf K., Muirhead V., Moynihan P., Weston-Price S., Pine C. (2018). Free Sugars Consumption around Bedtime and Dental Caries in Children: A Systematic Review. JDR Clin. Transl. Res..

[14] Crozier S.R., Robinson S.M., Borland S.E., Inskip H.M. (2006). Dietary patterns in the Southampton Women’s Survey. Eur. J. Clin. Nutr..

[15] Hu S., Sim Y.F., Toh J.Y., Saw S.M., Godfrey K.M., Chong Y.S., Yap F., Lee Y.S., Shek L.P.C., Tan K.H. (2019). Infant dietary patterns and early childhood caries in a multi-ethnic Asian cohort. Sci. Rep..

[16] Lim S., Tellez M., Ismail A.I. (2015). Dental caries development among African American children: Results from a 4-year longitudinal study. Community Dent. Oral Epidemiol..

[17] Poklepovic T., Worthington H.V., Johnson T.M., Sambunjak D., Imai P., Clarkson J.E., Tugwell P. (2013). Interdental brushing for the prevention and control of periodontal diseases and dental caries in adults. Cochrane Database of Systematic Reviews.

[18] Kumar S., Tadakamadla J., Johnson N.W. (2016). Effect of Toothbrushing Frequency on Incidence and Increment of Dental Caries: A Systematic Review and Meta-Analysis. J. Dent. Res..

[19] Marinho V.C.C., Higgins J.P.T., Logan S., Sheiham A. (2003). Topical fluoride (toothpastes, mouthrinses, gels or varnishes) for preventing dental caries in children and adolescents. Cochrane Database of Systematic Reviews.

[20] Wright J.T., Hanson N., Ristic H., Whall C.W., Estrich C.G., Zentz R.R. (2014). Fluoride toothpaste efficacy and safety in children younger than 6 years: A systematic review. J. Am. Dent. Assoc..

[21] Ellwood R.P., Cury J.A. (2009). How much toothpaste should a child under the age of 6 years use?. Eur. Arch. Paediatr. Dent..

[22] Hilgert L.A., Leal S.C., Mulder J., Creugers N.H.J., Frencken J.E. (2015). Caries-preventive Effect of Supervised Toothbrushing and Sealants. J. Dent. Res..

[23] Dos Santos A.P.P., de Oliveira B.H., Nadanovsky P. (2018). A systematic review of the effects of supervised toothbrushing on caries incidence in children and adolescents. Int. J. Paediatr. Dent..

[24] Melo P., Fine C., Malone S., Taylor S. (2021). Impact of the Brush Day & Night Programme on Oral Health Knowledge and Behaviour in Children. Int. Dent. J..

[25] Hall-Scullin E., Whitehead H., Milsom K., Tickle M., Su T.L., Walsh T. (2017). Longitudinal Study of Caries Development from Childhood to Adolescence. J. Dent. Res..

[26] Larsen P.S., Kamper-Jørgensen M., Adamson A., Barros H., Bonde J.P., Brescianini S., Brophy S., Casas M., Charles A.A., Devereux G. (2013). Pregnancy and birth cohort resources in Europe. A large opportunity for aetiological child health research. Paediatr. Perinat. Epidemiol..

[27] Vilela S., Severo M., Moreira T., Ramos E., Lopes C. (2019). Evaluation of a short food frequency questionnaire for dietary intake assessment among children. Eur. J. Clin. Nutr..

[28] Durão C., Andreozzi V., Oliveira A., Moreira P., Guerra A., Barros H., Lopes C. (2015). Maternal child-feeding practices and dietary inadequacy of 4-year-old children. Appetite.

[29] Valenzuela M.J., Waterhouse B., Aggarwal V.R., Bloor K., Doran T. (2021). Effect of sugar-sweetened beverages on oral health: A systematic review and meta-analysis. Eur. J. Public Health.

[30] Durão C., Severo M., Oliveira A., Moreira P., Guerra A., Barros H., Lopes C. (2017). Association of maternal characteristics and behaviours with 4-year-old children’s dietary patterns. Matern. Child Nutr..

[31] Byrt T. (1996). How Good Is That Agreement?. Epidemiology.

[32] Ismail A.I., Sohn W., Tellez M., Amaya A., Sen A., Hasson H., Pitts N.B. (2007). The International Caries Detection and Assessment System (ICDAS): An integrated system for measuring dental caries. Community Dent. Oral Epidemiol..

[33] Mattila M.L., Rautava P., Sillanpää M., Paunio P. (2000). Caries in five-year-old children and associations with family-related factors. J. Dent. Res..

[34] Leroy R., Bogaerts K., Lesaffre E., Declerck D. (2005). Multivariate survival analysis for the identification of factors associated with cavity formation in permanent first molars. Eur. J. Oral Sci..

[35] Winter J., Glaser M., Heinzel-Gutenbrunner M., Pieper K. (2015). Association of caries increment in preschool children with nutritional and preventive variables. Clin. Oral Investig..

[36] Ismail A.I., Sohn W., Lim S., Willem J.M. (2009). Predictors of dental caries progression in primary teeth. J. Dent. Res..

[37] Hoffmeister L., Moya P., Vidal C., Benadof D. (2016). Factors associated with early childhood caries in Chile. Gac. Sanit..

[38] Weber-Gasparoni K., Kanellis M.J., Levy S.M., Stock J. (2007). Caries prior to age 3 and breastfeeding: A survey of La Leche League members. J. Dent. Child.

[39] Featherstone J.D.B. (2004). The continuum of dental caries—Evidence for a dynamic disease process. J. Dent. Res..

[40] Llena C., Calabuig E. (2018). Risk factors associated with new caries lesions in permanent first molars in children: A 5-year historical cohort follow-up study. Clin. Oral Investig..

[41] Warren J.J., Blanchette D., Dawson D.V., Marshall T.A., Phipps K.R., Starr D., Drake D.R. (2016). Factors associated with dental caries in a group of American Indian children at age 36 months. Community Dent. Oral Epidemiol..

[42] Un Lam C., Khin L.W., Kalhan A.C., Yee R., Lee Y.S., Chong M.F.F., Kwek K., Saw S.M., Godfrey K., Chong Y.S. (2017). Identification of Caries Risk Determinants in Toddlers: Results of the GUSTO Birth Cohort Study. Caries Res..

[43] Wagner Y., Heinrich-Weltzien R. (2016). Evaluation of an interdisciplinary preventive programme for early childhood caries: Findings of a regional German birth cohort study. Clin. Oral Investig..

[44] Marinho A.R., Severo M., Correia D., Lobato L., Vilela S., Oliveira A., Ramos E., Torres D., Lopes C. (2020). Total, added and free sugar intakes, dietary sources and determinants of consumption in Portugal: The National Food, Nutrition and Physical Activity Survey (IAN-AF 2015–2016). Public Health Nutr..

[45] Calado R., Ferreira C.S., Nogueira P., Melo P. (2017). Caries prevalence and treatment needs in young people in Portugal: The third national study. Community Dent. Health.

[46] Direção-Geral da Saúde Programa Nacional de Promoção da Saúde Oral (2015). III Estudo Nacional de Prevalência das Doenças Orais.

[47] World Health Organization (1999). Health 21: The health for all policy framework for the WHO European Regions. European Health for All Series: World Health Organization.

