# Supplementary material for: Dietary Patterns and Oral Health Behaviours Associated with Caries Development from 4 to 7 Years of Age
Source: Life (Basel). 2021 Jun 24;11(7):609. doi: 10.3390/life11070609 (PMC8305377; doi:10.3390/life11070609)
Supplement: Supplementary file 1 [file life-11-00609-s001.zip › life-1252429-supplementary.pdf]

## Supplementary materials:

*Article*

# Dietary Patterns and Oral Health Behaviours Associated with Caries Development from 4 to 7 Years of Age

**Cátia Carvalho Silva [1,2,*], Sandra Gavinha [2], Sofia Vilela [3], Rita Rodrigues [2], Maria Conceição Manso [2,4,5], Milton Severo [3,6], Carla Lopes [3,6] and Paulo Melo [1,3]**

**Table S1.** Children's characteristics of the sample according to dental caries experience at 4 years of age ($d_{3-6}mft=0$ *vs.* $d_{3-6}mft>0$) and at 7-years of age ($d_{3-6}mft/D_{3-6}MFT=0$ *vs.* $d_{3-6}mft/D_{3-6}MFT>0$).

| | **Dental Caries Status** | | | | | |
|---|---|---|---|---|---|---|
| | **4 years of age** | | | **7 years of age** | | |
| | **$d_{3-6}mft=0$ n (%)** | **$d_{3-6}mft>0$ n (%)** | ***p****** | **$d_{3-6}mft/D_{3-6}MFT=0$ n (%)** | **$d_{3-6}mft/D_{3-6}MFT>0$ n (%)** | ***p****** |
| **Child's sex** | | | | | | |
| Female | 203 (46.1) | 86 (51.5) | 0.238 | 138 (49.6) | 151 (45.9) | 0.358 |
| Male | 237 (53.9) | 81 (48.5) | | 140 (50.4) | 178 (54.1) | |
| **Maternal education** | | | | | | |
| ≤ 9 years | 177 (40.4) | **82 (49.1)** | **0.004** | 94 (33.8) | **165 (50.5)** | **<0.001** |
| 10-12 years | 126 (28.8) | 56 (33.5) | | 87 (31.3) | 95 (29.1) | |
| >12 years | **135 (30.8)** | 29 (17.4) | | **97 (34.9)** | 67 (20.5) | |
| **Monthly household income** | | | | | | |
| Lower: ≤1000€ | 92 (21.2) | 71 (43.0) | **<0.001** | 51 (18.5) | 112 (34.6) | **<0.001** |
| Intermediate: 1001-1500€ | 121 (27.9) | 44 (26.7) | | 68 (24.7) | 97 (29.9) | |
| Higher: >1500€ | 221 (50.9) | 50 (30.3) | | 156 (56.7) | 115 (35.5) | |
| **Toothbrushing frequency** | | | | | | |
| < 2 or ≥ 2 times per day, none at bedtime | 194 (44.7) | 81 (49.0) | 0.306 | 114 (41.5) | 161 (49.8) | **0.040** |
| ≥ 2 times per day, 1 at bedtime | 240 (55.3) | 83 (50.6) | | 161 (58.5) | 162 (50.2) | |
| **Fluoride toothpaste** | | | | | | |
| No | 1 (0.2) | 1 (0.6) | 0.469 | 12 (5.8) | 17 (7.0) | 0.607 |
| Yes | 434 (99.8) | 161 (99.4) | | 194 (94.2) | 225 (93.0) | |
| **Parental-supervised toothbrushing** | | | | | | |
| No | 269 (64.4) | 131 (82.9) | **<0.001** | 256 (93.8) | 288 (88.3) | **0.022** |
| Yes | 149 (35.6) | 27 (17.1) | | 17 (6.2) | 38 (11.7) | |
| **Dental appointment at 4-year-old** | | | | | | |
| No | 314 (71.9) | 88 (52.7) | **<0.001** | 188 (68.4) | 214 (65.0) | 0.389 |
| Yes | 123 (28.1) | 79 (47.3) | | 87 (31.6) | 115 (35.0) | |
| **Eating before go to bed** | | | | | | |
| No | 252 (58.3) | 90 (54.5) | 0.403 | 169 (62.4) | 173 (53.1) | **0.022** |
| Yes | 180 (41.7) | 75 (45.5) | | 102 (37.6) | 153 (46.9) | |
| **Dietary patterns** | | | | | | |
| Energy-dense foods | 189 (43.5) | 85 (51.5) | **0.048** | 107 (38.8) | 167 (51.7) | **0.002** |
| Snacking | 55 (12.7) | 26 (15.8) | | 36 (13.0) | 45 (13.9) | |
| Healthier | 190 (43.8) | 54 (32.7) | | 133 (48.2) | 111 (34.4) | |
| **Daily frequency of cariogenic food intake** | | | | | | |
| n | 325 | 118 | 0.822 | 204 | 239 | 0.349 |
| Median (IQR) | 2.76 (1.98-3.84) | 2.81 (1.72-4.23) | | 2.72 (2.01-3.68) | 2.82 (1.84-4.04) | |

| | Dental Caries Status | | | | | |
|---|---|---|---|---|---|---|
| | 4 years of age | | | 7 years of age | | |
| | **$d_{3-6}mft=0$ n (%)** | **$d_{3-6}mft>0$ n (%)** | ***p**** | **$d_{3-6}mft/D_{3-6}MFT=0$ n (%)** | **$d_{3-6}mft/D_{3-6}MFT>0$ n (%)** | ***p**** |
| **Daily frequency of cariogenic drinks intake** | | | | | | |
| n | 326 | 118 | **0.001** | 205 | 239 | **0.020** |
| Median (IQR) | 0.67 (0.26-1.26) | 0.98 (0.42-1.98) | | 0.65 (0.26-1.26) | 0.87 (0.34-1.48) | |

Bold entries denote statistical significance ($p<0.05$); *$p$-value: Mann-Whitney U-test, chi-square test or Fisher's Exact test, as appropriate; $p<0.001$ (Bonferroni correction); dmft, decayed missing and filled primary teeth; DMFT: decayed missing and filled permanent teeth; IQR, Interquartile Range.
